# The Effect of COVID-19 on Middle-Aged Adults’ Mental Health: A Mixed-Method Case–Control Study on the Moderating Effect of Cognitive Reserve

**DOI:** 10.3390/healthcare12020163

**Published:** 2024-01-10

**Authors:** Barbara Colombo, Giulia Fusi, Kenneth B. Christopher

**Affiliations:** 1 Behavioral Neuroscience Lab, Champlain College, Burlington, VT 05401, USA; 2 Department of Human and Social Sciences, University of Bergamo, 24129 Bergamo, Italy; giulia.fusi@unibg.it; 3 Channing Division of Network Medicine, Brigham and Women’s Hospital, Harvard Medical School, Boston, MA 02115, USA; kbchristopher@bwh.harvard.edu; 4 Division of Renal Medicine, Brigham and Women’s Hospital, Harvard Medical School, Boston, MA 02115, USA

**Keywords:** COVID-19, cognitive reserve, PTSD, mental health, text analysis

## Abstract

The COVID-19 pandemic has increased the vulnerability of adults to mental health effects, and the study of protective factors has become crucial. Cognitive reserve (CR) is a well-known protective factor against cognitive decline and several health factors; however, its protective effect on mental health during the pandemic has been rarely addressed. Thus, this study explored, through a mixed-method design, the effect of CR on perceived distress and PTSD-like symptoms in middle-aged participants who have survived severe COVID-19 and a matched control group. A total of 432 participants filled out self-report measures of CR, PTSD, depression, and anxiety, and were also asked to provide narration about their COVID-19-related experience. COVID-19 significantly affected the chances of reporting different mental health symptoms; levels of CR played a protective role in reducing their severity. Moreover, adults with higher CR seemed to be more realistic, focusing less on positive emotions, and elaborating more on the sense of anxiety when describing their experience: this might be an indication of a lower use of suppression to regulate emotions. Practical implications of these findings and future directions have been also discussed.

## 1. Introduction

As of September 2023, there were almost 770 million confirmed cases of COVID-19 and 6.9 million associated deaths worldwide. It is now recognized [1,2,3] that some COVID-19 survivors experience chronic complications that result from the treatments as well as from related difficulties that often arise, both on a physiological and psychological level. Focusing on mental health consequences reported in patients who recover from COVID-19, recent meta-analyses [1,4,5] highlight how the pooled prevalence of mental health-related symptoms was 26%. This included a high incidence of symptoms related to post-traumatic stress disorder (PTSD), with a pooled prevalence of 21.94–33.00%, alongside anxiety (15.50–28.00%), stress (13.29–27.00%), and depression (15.97–22.00%). Another recent meta-analysis [6] reported similar data, emphasizing the high level of PTSD-like symptoms and depression displayed by COVID-19 patients. 

More worrisome is that similar mental health consequences, although generally less severe, have been reported for the general adult population who have been exposed to COVID-19 risks and COVID-19-related protective measures [7]. 

The pandemic might have increased the vulnerability of adults to the mental health effects of COVID-19 due to the additional stress derived from prolonged social isolation [8,9], work insecurity [10,11], and perceived health risks [12,13]. This general effect on the adult population could be expected to be more pronounced in middle-aged adults, who have been reported to begin exhibiting psychological and physiological patterns in response to stress similar to those of the aging population [14], a process that might have been enhanced by the pandemic. Thus, it is relevant to explore the protective factors of mental health effects of COVID-19 in middle-aged adults. Social support, adaptive cognitive emotion regulation [15,16], optimism, and resilience [16,17] are all noted to possibly mitigate the mental health effects of COVID-19. Cognitive reserve (CR), a process that leads to the accumulation of brain resources during the entire lifespan [18,19], may be related to such social [20], emotional [21], and cognitive [22] protective factors, which could be applied to both the aging and middle-aged population. 

The CR Is considered a protective factor for the aging population, both for cognitive performance and promotion of overall well-being [23,24,25]. The CR model suggests that the flexibility and adaptability of brain networks might allow the brain to actively resist the effects of age- or disease-related changes and to therefore cope with brain damage [19,26,27,28]; in addition, it can also sustain individuals to choose the right cognitive and emotional strategies [21,29] that can promote psychological resilience, which is correlated with higher well-being and quality of life [30]. CR is commonly measured by using proxies—such as educational level [31,32], occupation type, and level of complexity [33,34]—and the level of engagement in cognitively based leisure activities [35,36]. These proxies have also been integrated into ad hoc tests [23,37] with the idea that the higher the CR levels are, the more that subjects can adapt to age- or disease-related modifications. These characteristics make CR a good candidate to be a possible protective factor from the adverse psychological effects of COVID-19, both for the elderly and for younger populations [38].

Some studies have indeed already shown in other countries that cognitive reserve appears to be a protective factor following COVID-19 infection, both in terms of risk of cognitive performance impairment [38] and the perception of psychological distress [39,40,41,42]. Starting from the evidence and theoretical background discussed above, and to deepen this line of study, we wanted to focus on how much CR can be also a precursor and a protective factor in nonelderly populations [43,44,45,46].

Therefore, this study explores the possible protective effects of CR on perceived distress, on the emergence of PTSD-like symptoms, and on psychological distress in healthy middle-aged subjects (>54 years) who have survived a severe case of the COVID-19 virus (which required hospitalization) and a control group of adults matched to our sample by age, gender, and education level. 

Moreover, to gain deeper insight into the overall cognitive and emotional personal experience of COVID-19 patients, a software-based text analysis approach was utilized. Several studies. Refs. [47,48,49] have used a text analysis approach to examine media-based, social media, or political communications about COVID-19 on patients’ narrations but research is still scarce and focuses only on specific categories of patients, such as cancer patients [50] or patients with autoimmune disease [51]. What is lacking is a focus on the cognitive and emotional personal experience of COVID-19 patients overall. We believe that this approach would be potentially relevant because it might help to highlight and confirm specific emotion regulation mechanisms in patients who have higher CR levels, providing a better understanding of the specific role that CR plays as a protective factor. We hypothesized that one’s CR might have a protective influence on the emergence of symptoms related to psychological distress (anxiety, stress, and depression levels) and PTSD-like symptoms (intrusive thoughts, flashbacks, increased arousal, or avoidance behaviors). 

Our study aimed to explore the mental health effects of contracting severe COVID (defined as a form of COVID-19 presenting symptoms that prevented the individual from performing normal daily life activities and required hospitalization) on middle-aged individuals by also considering the possible protective effects of the individuals’ CR. Considering the study results to date:

1. We expect to find a significant negative effect of COVID-19 on the severity of PTSD-like symptoms and levels of reported depression, anxiety, and stress;
2. We expect psychological symptoms to be significantly reduced by the individuals’ CR levels;
3. We expect individuals who had COVID-19 and recovered to describe their experience differently depending on their CR level, using indicators of better emotional regulation strategies when they have a higher CR level.

## 2. Materials and Methods

This study was approved by Champlain College IRB (COA IRB000173). Participants were recruited through social media (Facebook) and word-of-mouth and were asked to fill out three self-report surveys on Qualtrics. Recruitment ads asked the people interested in sharing their experience of COVID-19 to get in touch with researchers to check for eligibility. Interested participants were screened for eligibility criteria and were assigned to the case or control group as detailed below. Data were collected after the first wave of the pandemic, starting in October 2020 and continuing until early spring of 2021.

Participants who reported contracting COVID-19 and experiencing symptoms that prevented them from performing normal activities of daily life and required hospitalization were asked to write a short narration describing their experience with COVID-19 and were assigned to the “cases” group, while participants who reported having contracted COVID-19 but who experienced no symptoms or only mild symptoms that did not prevent them from executing their daily activities (e.g., cold-like symptoms) and did not require hospitalization were excluded from the study. Individuals who reported to have never tested positive for COVID-19 and never experienced COVID-19-like symptoms in the previous months were recruited to the control group.

### 2.1. Sample

We recruited participants by posting flyers in local hospitals, health centers, and family medicine practices. To be more likely to recruit enough control participants, we posted fliers in local community centers, coffee bars, and supermarkets. People who were interested were screened to be sure that they met the recruitment criteria (including self-reporting no medical history of mental health problems, e.g., depression or anxiety disorders diagnosed before the COVID-19 pandemic).

A total of 432 participants joined this study. Half of the participants (216) were infected by the COVID-19 virus during the first lockdown and recovered. The other half of the sample were healthy controls, matched by age, gender (F = 143 for both groups), and level of education. Demographic information about the sample is reported in Table 1.

### 2.2. Materials

An integrated assessment of the CR, the CoRe-T [23], was selected for this study. Apart from combining all of the traditionally used proxies (see Introduction section), the CoRe-T also has the advantage of measuring the flexibility of thought, which is and has been reported to be positively correlated with the CR both in healthy [52,53,54] and clinical populations [55,56,57,58]. The CoRe-T has two main sections: self-report and fluidity of thoughts tasks. Self-report data included information about education level such as years of completed education, including vocational training; type, frequency, and longevity of leisure activities; and occupation history such as type of occupations and the number of years they have been working in each position. Two tasks are used to assess fluidity of thought: the acronyms task, in which participants are given five minutes to list all the terms that can fit into three given acronyms (the terms had to make sense together), and the alternative-uses task, where participants are asked to list as many different, interesting, or unusual uses for an empty plastic bottle as they can in five minutes. Scoring procedures are described in detail in [23].

The revised version of the Impact of Event Scale (IES-r) [59] is a self-report measure of current subjective distress in response to a specific traumatic event. It comprises three subscales representative of the major symptom clusters of post-traumatic stress: intrusion (i.e., nightmares, unbidden visual images of the trauma while awake, intrusive thoughts about aspects of the traumatic event, sequelae, or self-conceptions), avoidance (i.e., subjects’ deliberate efforts to not think or talk about the traumatic event and to avoid reminders of the event), and hyper-arousal (i.e., symptoms of greater anger, irritability, jumpiness and exaggerated startle response, trouble concentrating, hypervigilance). Total scores of PTSD ranging from 0 to 88 were also calculated, with higher scores indicating a greater probability of the presence of PTSD. Scores of 24 or higher are linked to a clinical concern for possible PTSD. In the current study, we modified the instructions, asking our participants to focus on their experiences linked to COVID-19 while answering the questions.

The 42-item Depression Anxiety Stress Scale (DASS) [60,61] was used to assess stress, anxiety, and depression levels. This scale provides a general distress score and three subscale scores of stress, anxiety, and depression. Participants must respond to each item on a 4-point Likert scale defined as follows: 0 = it has never happened to me; 1 = it has happened to me a few times; 2 = it has happened to me quite frequently; and 3 = it happens to me almost always. Example items are the following: “I felt stressed”; “I felt very fatigued with difficulty in breathing, for example, very fast breathing, feeling very fatigued without physical effort”; and “I see nothing good in my future”. The scale was chosen because of its suitability with both clinical and non-clinical populations [62] and its focus on both stress and anxiety.

Finally, as mentioned above, participants who recovered from COVID-19 were also asked to write a short description of their experience with the virus using their own words. These narrations were analyzed using linguistic inquiry and word count (LIWC-22) software [63]. This software analyzes the content of a text in a sequential way, by matching each word with a reference dictionary organized by categories, such as emotional language, which includes words that refer to emotions, or time orientation, which includes words and verb tenses that imply a focus on the past, the present, or the future. When a match is found for a category or categories, meaning that the same words can be included in more than one category, that specific score is incremented, generating a score for the target variable. The values that the software returns represent the mean percentages of the words of the examined text that fall into the target category. For example, a mean score of 5.32 for cognitive processes means that 5.32% of the words used by participants were linked to cognitive processes. We selected our target categories based on studies that used them to investigate the same variables [64,65,66]. A detailed description of LIWC categories used in this study—with examples of the words belonging to each category and more examples of previous relevant studies that have used these variables to explore variables similar to the ones considered in this study—can be found in the Supplementary Materials.

### 2.3. Statistical Analyses

Analyses were run using Stata 17 (USA), and all variables were normally distributed. Thus, a linear regression model was run to examine how having or not having had COVID-19, the independent variable, would affect the chances of developing PTSD or symptoms such as depression, anxiety, and stress. To reduce the effects of confounding we used propensity scores. Propensity scores represent a robust statistical method developed to address confounding in case–control studies, ensuring comparability between treated and untreated subjects when randomization is not feasible. Rosenbaum and Rubin [67] introduced the propensity score as the conditional probability of assignment to a particular treatment given a vector of observed covariates. Propensity-score matching has long been utilized in social science and clinical research to account for confounding [68,69]. The Stata command psmatch2 [70] was used to match cases and controls according to their propensity scores, using the caliper = 0.005—a caliper is a predefined width around the propensity score of each individual in the treated group (or vice versa) within which a control is considered an acceptable match. By setting a caliper, we imposed a limit on the maximum allowable distance between the propensity scores of matched treated and control units, ensuring that matches are made between units with similar propensity scores. Ensuring closeness in the propensity scores between treated and control subjects improves the quality of the matched sample and increases the validity of subsequent analyses that rely on the matched pairs. The total IES score was set as an outcome variable, while age, gender, and total years of education were used as covariates. Eighty-eight cases were dropped based on propensity scores. Finally, the narrations provided by COVID-19 patients were analyzed using LIWC software. Descriptive analyses were provided to observe and describe COVID-19 patients’ narration characteristics, and a linear regression model was run with CR scores as predictors to explore the specific role of patients’ CR in predicting the use of our target categories (e.g., general affect, negative emotions, anxiety, etc.; see Section 2.2).

## 3. Results

As a first step, we worked with our full sample, examining how having had COVID-19 would be related to one’s chances of developing PTSD or depression, anxiety, and stress (Hp1). Mean scores are reported in Table 2.

The results revealed that having or not having had COVID-19 significantly affected the chances of developing symptoms related to PTSD, depression, anxiety, and stress (Hp 1). See Table 3 for details.

When adding the CR score into the model (see Table 4. Hp. 2), we found that the relationship between having been infected with COVID-19 and the chances of developing PTSD or depression was still significant but CR also had a significant effect in both models. Since the coefficient of the CR score was negative, we can assume that the higher the CR index was, the lower the PTSD symptomatology; CR, therefore, played a significant protective role.

The second step included the use of the previously cited Stata command to match cases and controls according to their propensity scores and the total IES score as an outcome variable (Hp 1). The covariates used in the model were age, gender, and total years of education. We ran the regression models again, obtaining the same significant results (see Table 5 and Table 6).

**Figure 1:**
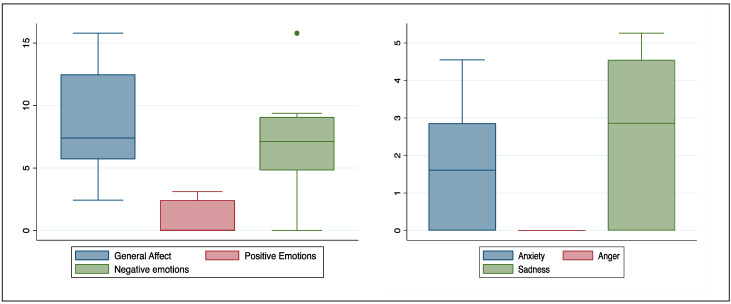
Use of emotional language.

As a third step, we analyzed the narration provided by COVID-19 patients using LIWC software (hp. 3). The first focal point of interest was the use of emotional language within the narration. Figure 1 depicts the distribution of patients’ use of a general tone, a positive or a negative tone. Since there was a predominance of negative emotions, we explored specific negative emotions such as anxiety, sadness, and anger in more depth, and the distribution is also reported in Figure 1.

In our sample, the emotion of sadness seemed to be predominant when patients were reminiscing about their experience with COVID-19. Interestingly, anger was not part of their narration.

**Figure 2:**
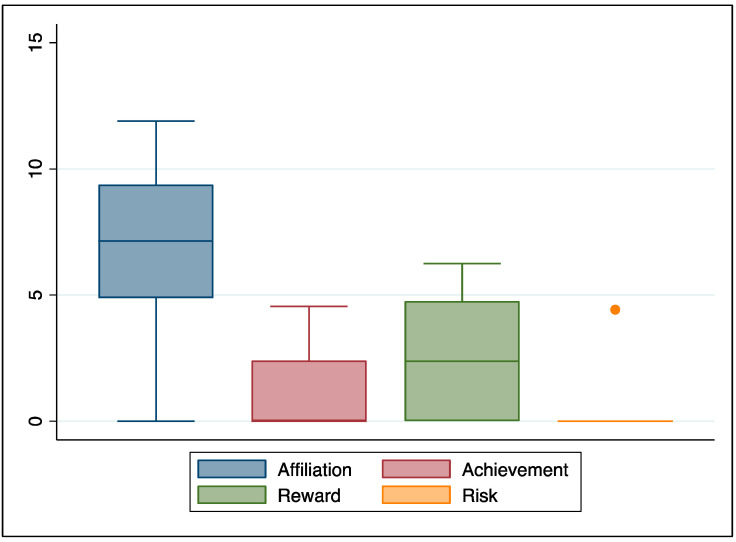
Drive-related language.

We also explored which drives could be derived from the analysis of their narrations, and the distributions are presented in Figure 2. Patients seemed to be most driven by the need for affiliation. Interestingly, our patients did not seem to be risk-takers.

**Figure 3:**
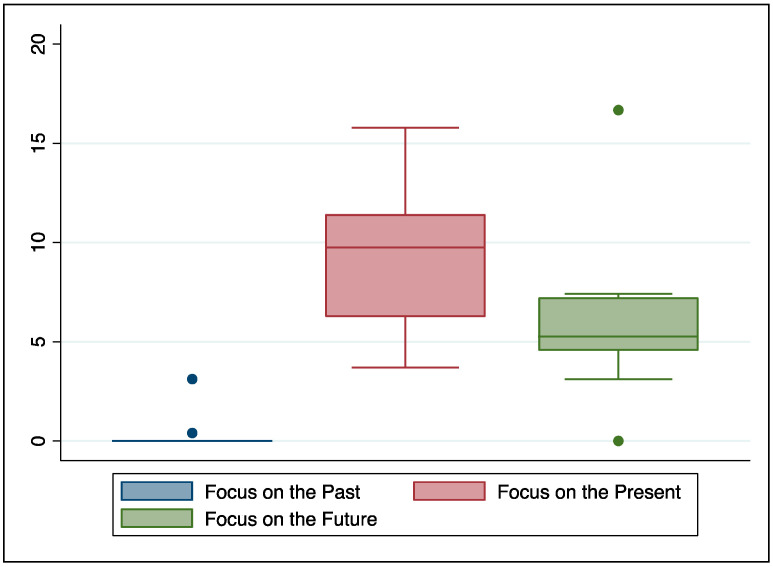
Time orientation as derived by patients’ language use.

Finally, we focused on patients’ time orientation while narrating their COVID-19 experience. As can be seen in Figure 3, patients appeared to be focused mainly on the present.

To be able to answer our research question (hp 3), we used linear regression models to explore the specific role of patients’ CR in predicting the use of our target categories. In the basic model, we used the total CR score as a predictor and language variables as outcomes (Table 7). Higher levels of CR seem to significantly predict an increased focus on discussing anxiety, a need for achievement, and a stronger focus on the present and future. Conversely, patients with lower CR focused less on other emotions and engaged in less discussion of the need for affiliation or risk-taking.

In the follow-up models we added the IES and DASS scores as possible confounders to the model but the significance of CR as a predictor did not change (Table S2—provided as part of the Supplementary Materials). We did not run the second regression for the emotion of anger, since, as indicated in Table 7 and Figure 1, no one used anger-related words in their narration.

## 4. Discussion

This study explored the effects of COVID-19 on middle-aged individuals’ mental health, comparing self-report data from patients who had a severe case of COVID-19 and recovered with a matched control group who never had COVID-19.

Our first hypothesis was that the total sample would have experienced PTSD and depressive symptoms but that these would be more pronounced in patients who recovered from COVID-19. Our results confirmed this hypothesis. These data support other findings reported in the literature, which notably focused only on problems detected in patients [71,72,73] or in non-infected/asymptomatic individuals who were affected only by the restrictions linked to the lockdown [74,75,76]. As far as we know, there are two studies that compared the mental health consequences of COVID-19 for both infected and non-infected/asymptomatic individuals. The first is a scoping review [6], which compares results from different studies rather than results from two matched samples. The second one [77] focuses on the seroprevalence of COVID-19, and while it has the added value of not excluding asymptomatic patients (as could be the case for a self-report study), it has a different focus: examining the effect of a pre-existing condition (a variable that we were not examining and we controlled for with our exclusion criteria), and has an interest in the effect of the COVID-19 virus rather than the COVID-19-related experience. Our study, by comparing patients who had been hospitalized for severe COVID-19 to individuals who reported no symptoms, focused more on the psychological effect of a severe physical illness.

By directly comparing two equivalent and matched samples of infected and non-infected/asymptomatic individuals, our study allows not only a confirmation of the fact that both groups experienced a significant mental health toll because of the pandemic but that consequences for the infected population were significantly higher, allowing a more precise estimate of the effects of COVID-19 on various mental health symptoms, in particular on PTSD and anxiety.

Our study added a further step to the survey of mental health consequences looking for possible protective factors. Specifically, we focused on the possible protective role of individuals’ CR, expecting that higher levels of CR would have been a protective factor against both PTSD and depressive symptoms. This second hypothesis was also confirmed. CR protected both samples in a significant way, reducing the severity of both PTSD and depressive symptoms. This result has never been reported for COVID-19 patients but the protective role of CR toward some PTSD symptoms among the middle-aged population has been reported in the literature in a study on 9/11 survivors [78]. How can we explain this effect? Rakesh, Morey [79] introduced the concept of a “resilience reserve”, defining it as a construct analogous to and overlapping with Stern’s CR, and used it to summarize the total physiological processes that protect and compensate specifically for the effect of trauma. The fact that we were able to identify this effect using the traditional proxies used to measure CR provides more evidence to support the concept and positive role of the resilience reserve and strengthens the association and overlap between the resilience reserve and CR. This also suggests that CR measures could be useful when assessing PTSD patients and that interventions aimed at increasing CR could potentially increase mental health in individuals exposed to traumatic events, such as the pandemic or a life-threatening illness, something that has been tested in a recent study [24].

Having established the promising role of the CR, our third aim was to use a more in-depth approach to analyze individuals’ personal narration of their experience with COVID-19 to highlight exactly which aspects of that traumatic experience were influenced the most by their CR. To be more specific, we hypothesized that infected individuals would describe their experience differently depending on their CR level, using indicators of better emotional regulation strategies when they have a higher CR level. Results seem to confirm this vision. Individuals with a higher CR seemed to be more realistic, focusing less on positive emotions when describing their experience with COVID-19, and elaborating more on the sense of anxiety that they experienced. This can be read as an indication that the higher CR led these individuals to use suppression less to regulate the negative emotions linked to the traumatic experience of having COVID-19. Suppression is a commonly used mechanism to manage a negative experience but it has been reported to be rather ineffective and to lead to memory distortion [80]. In our case, the memory distortion could possibly be identified in the higher focus on positive emotion in the narration of individuals with lower CR.

Another interesting result linked to emotion regulation is the fact that patients with higher CR also focused significantly more on the present. Being mindful has been reported to be associated with higher positive affect and to be negatively related to emotion suppression [81]. These patients also focused more on the future, a factor that has been linked to better emotion regulation and higher levels of well-being in individuals affected by a COVID-19 lockdown [82].

Finally, a higher level of CR also promoted different drives in our patients: those with a higher CR focused more on a sense of achievement and affiliation. A sense of achievement has been suggested to constitute part of one’s psychological well-being [83] and has also been listed as a factor that contributes significantly to success with interventions aimed at increasing well-being in clinical settings [84]. This specific result can be used to better understand the reasons behind the higher level of psychological well-being—derived by lower levels of PTSD, anxiety, depression, and stress—reported by individuals with a higher CR. The higher scores related to the sense of affiliation can be read in a similar way since a higher sense of affiliation and belonging has been reported to increase the well-being of older individuals [20,85,86,87] as well as generally for adults [88,89] and clinical populations [90,91,92,93].

Our study has several strengths. An aspect that might be missing from previous studies is a deeper insight into patients’ perspectives as well as a broader look at other individuals’ perspectives. We believe the software-based text analysis approach is relevant to better understand how CR may be protective. This assumption, derived from narrative inquiry [94] and narrative medicine [95,96], relies on previous studies that were able to use text analysis to derive specific indicators of reappraisal [97,98,99]. This approach has also been used by scholars in psychological and clinical sciences to analyze the cognitive, emotional, and structural components behind natural language [100]. It has also been successfully used in COVID-19-related research—for example, analyzing social media conversations [101] or the content of dreams [102]—but no one, to our knowledge, has used it in a case–control study to support the findings provided by other quantitative data.

This study also presents some limitations that should be addressed in future studies. First, premorbid psychiatric conditions were not controlled in this study other than by asking participants to self-declare that they had no history of previous mental health conditions. The sample size of this study is large enough to rule out the possibility that the data are invalidated by this finding but it is nonetheless something to consider for the generalizability of the present results. The self-report nature of our data also poses a limitation in distinguishing between COVID-19-infected, non-COVID-19-infected, and asymptomatic patients, yet our focus was more on comparing the experiences of those who were hospitalized for severe COVID-19 to those who reported no symptoms, so our data are still relevant to the aim of this study. The possible effect of different COVID-19 variants was not explored because this info was not provided by our patients. Finally, there is a lack of follow-up data that might better disentangle how long these psychological symptoms will last both for COVID-19 patients and for the general population.

## 5. Conclusions

This study presents an interesting comparison between a sample of middle-aged American adults who contracted a serious case of COVID-19 and recovered with a matched sample of healthy controls. We were able to demonstrate with a direct comparison how much more severe the effect of COVID-19 on mental health has been for patients. We were also able to identify a possible protective factor, an individual’s level of CR. A higher CR seems to promote better emotion regulation strategies and support a sense of achievement and belonging, which, in turn, might play a role in increasing psychological well-being. Data from this study could be used as a starting point to build interventions to support COVID-19 survivors.

Future studies should use a longitudinal design to record possible changes over time, check for the effect of cultural differences, and attempt to replicate the findings working with different age groups (e.g., older adults) and vulnerable populations who are affected by different types of traumas.

The results emerging from this study might inform health practitioners working with long-COVID patients and suggest working on emotion regulation strategies, on top of using a narrative medicine approach. Focusing on programs to increase CR could also prove to be beneficial, as was reported in studies conducted on the general population [24], and should also be tested in future research with long-COVID patients.

## Figures and Tables

**Table 1:** Demographic information for COVID-19 patients and control group.

	Patients *N* = 216	Controls *N* = 216	95% CI 95% Confidence Intervals
Age	Range: 54–66 Mean: 58.32 SD: 4.21	Range: 54–65 Mean: 58.14 SD: 4.14	−1.02–0.55
Years of completed education	Range: 10–27 Mean: 17.25 SD: 4.25	Range: 10–25 Mean: 16.92 SD: 4.24	−1.09–0.44

**Table 2:** Mean scores and standard deviation of the main study variables, by condition.

Variable	Min–Max	Mean	Std. Dev.
Controls			
PTSD—Total score	14–66	34.32	13.26
Intrusion	0.39–2.89	1.51	0.65
Avoidance	0.28–3	1.35	0.64
Hyperarousal	0.21–3	1.40	0.62
Depression	10–37	18.04	6.14
Anxiety	11–33	16.92	6.05
Stress	10–29	17.70	6.14
Cognitive reserve	45–106	76.12	13.43
Cases			
PTSD—Total score	19–69	40.76	15.44
Intrusion	1–3	1.92	0.72
Avoidance	1–3	1.81	0.69
Hyperarousal	1–3	1.80	0.68
Depression	11–40	20.87	7.38
Anxiety	12–39	20.05	7.63
Stress	11–36	20.42	7.38
Cognitive reserve	44–122	81.07	17.19

**Table 3:** Linear regression model—effect of COVID-19 status on mental health.

	β Coefficient	*p*	95% CI
IES			
COVID status _PTSD total score_	6.43	<0.0001	3.71–9.15
COVID status _Intrusion_	0.41	<0.0001	0.28–0.54
COVID status _Avoidance_	0.45	<0.0001	0.32 –0.58
COVID status _Hyperarousal_	0.40	<0.0001	0.27–0.52
DASS			
COVID status _Depression_	2.83	<0.0001	1.55–4.12
COVID status _Anxiety_	3.12	<0.0001	1.82–4.43
COVID status _Stress_	2.72	<0.0001	1.51–3.94

**Table 4:** Linear regression model controlling for the effect of cognitive reserve on post-traumatic stress disorder symptoms, depression, anxiety, and stress.

	β Coefficient	*p*	95% CI
IES—PTSD, total			
COVID positive/negative	9.85	<0.0001	7.98–11.71
Cognitive reserve	−0.69	<0.0001	−0.75–−0.63
IES—Intrusion			
COVID positive/negative	0.57	<0.0001	0.47–0.66
Cognitive reserve	−0.03	<0.0001	−0.04–−0.03
IES—Avoidance			
COVID positive/negative	0.61	<0.0001	0.53–0.70
Cognitive reserve	−0.03	<0.0001	−0.04–−0.03
IES—Hyperarousal			
COVID positive/negative	0.55	<0.0001	0.47–0.64
Cognitive reserve	−0.03	<0.0001	−0.04–−0.03
DASS—Depression			
COVID positive/negative	4.40	<0.0001	3.50–5.30
Cognitive reserve	−0.32	<0.0001	−0.35–−0.29
DASS—Anxiety			
COVID positive/negative	4.98	<0.0001	4.26–5.70
Cognitive reserve	−0.37	<0.0001	−0.40–−0.35
DASS—Stress			
COVID positive/negative	4.03	<0.0001	3.09–4.98
Cognitive reserve	−0.26	<0.0001	−0.30–−0.23

**Table 5:** Linear regression model controlling for the effect of COVID-19 status on mental health—matched sample.

	β Coefficient	*p*	95% CI
IES			
COVID positive/negative _PTSD total score_	5.29	<0.0001	2.33–8.25
COVID positive/negative _Intrusion_	0.33	<0.0001	0.19–0.47
COVID positive/negative _Avoidance_	0.35	<0.0001	0.21–0.49
COVID positive/negative _Hyperarousal_	0.33	<0.0001	0.19–0.46
DASS			
COVID positive/negative _Depression_	1.46	0.038	0.08–2.85
COVID positive/negative _Anxiety_	1.91	0.009	0.47–3.34
COVID positive/negative _Stress_	1.90	0.005	0.58–3.22

**Table 6:** Linear regression model controlling for the effect of the cognitive reserve on post-traumatic stress disorder symptoms, depression, anxiety, and stress—matched sample.

	β Coefficient	*p*	95% CI
IES—PTSD, total			
COVID status	9.05	<0.0001	7.00–11.11
Cognitive reserve	−0.68	<0.0001	−0.74–−0.61
IES—Intrusion			
COVID status	0.50	<0.0001	0.40–0.61
Cognitive reserve	−0.03	<0.0001	−0.04–−0.03
IES—Avoidance			
COVID status	0.53	<0.0001	0.44–0.63
Cognitive reserve	−0.03	<0.0001	−0.04–−0.03
IES—Hyperarousal			
COVID status	0.50	<0.0001	0.41–0.59
Cognitive reserve	−0.03	<0.0001	−0.04–−0.03
DASS—Depression			
COVID status	3.22	<0.0001	2.26–4.19
Cognitive reserve	−0.32	<0.0001	−0.35–−0.29
DASS—Anxiety			
COVID status	4.00	<0.0001	3.20–4.79
Cognitive reserve	−0.38	<0.0001	−0.41–−0.35
DASS—Stress			
COVID status	3.32	<0.0001	2.26–4.38
Cognitive reserve	−0.26	<0.0001	−0.29–−0.22

**Table 7:** Linear regression model—effect of cognitive reserve on language variables.

Effect of Cognitive Reserve on:	β Coefficient	*p*	95% CI
General Affect	−0.04	0.04	−0.07–−0.001
Positive Emotions	−0.02	<0.0001	−0.04–−0.01
Negative Emotions	−0.03	0.07	−0.06–0.01
Anxiety	0.05	<0.001	0.03–0.06
Anger	omitted	omitted	omitted
Sadness	−0.07	<0.001	−0.08–−0.05
Affiliation	0.12	<001	0.10–0.15
Achievement	0.03	<0.001	0.02–0.04
Reward	0.02	0.07	−0.01–0.04
Risk-taking	−0.05	<0.001	−0.05–−0.04
Focus on the past	−0.01	0.05	−0.02–0.001
Focus on the present	0.13	<0.001	0.10–0.16
Focus on the future	0.19	<0.001	0.16–0.21

## Data Availability

Data are available upon request by writing to the corresponding author.

## Supplementary Materials

The following supporting information can be downloaded at: https://www.mdpi.com/article/10.3390/healthcare12020163/s1, Table S1: Description of LIWC categories used in this study with examples of the words belonging to each category and examples of previous relevant studies. Table S2: Follow-up regression models with added predictors (IES and DASS scores). Refs. [103,104,105,106] are cited in Supplementary Materials.
