# Supplementary material for: The Effect of COVID-19 on Middle-Aged Adults’ Mental Health: A Mixed-Method Case–Control Study on the Moderating Effect of Cognitive Reserve
Source: Healthcare (Basel). 2024 Jan 10;12(2):163. doi: 10.3390/healthcare12020163 (PMC10815714; doi:10.3390/healthcare12020163)
Supplement: Supplementary file 1 [file healthcare-12-00163-s001.zip › healthcare-2788460-supplementary.pdf]

Table S1. Description of LIWC categories used in the study with examples of the words belonging to each category and examples of previous relevant studies.

**Category**<br><br><u>*Comprehensive categories*</u><br>Sub-categories	**Examples**	**Examples of previous relevant studies that used this category**
<u>*Summary Language Variables*</u><br><br><u>*(Studies quoted here used all the sub- categories listed below)*</u>		Lumontod III, R. Z. (2020). Seeing the invisible: Extracting signs of depression and suicidal ideation from college students' writing using LIWC a computerized text analysis. *Int. J. Res. Stud. Educ*, *9*, 31-44.<br><br>Low, D. M., Rumker, L., Talkar, T., Torous, J., Cecchi, G., & Ghosh, S. S. (2020). Natural language processing reveals vulnerable mental health support groups and heightened health anxiety on reddit during covid-19: Observational study. *Journal of medical Internet research*, *22*(10), e22635.
Analytical Thinking	/	
Clout	/	
Emotional tone	/	
<u>*Linguistic dimensions*</u><br><br><u>*(Studies quoted here used all the sub- categories listed below)*</u>		Lumontod III, R. Z. (2020). Seeing the invisible: Extracting signs of depression and suicidal ideation from college students' writing using LIWC a computerized text analysis. *Int. J. Res. Stud. Educ*, *9*, 31-44.
Personal pronouns	I, them, her	
1st person singular	I, me, mine	Robertson, S. M., Short, S. D., Sawyer, L., & Sweazy, S. (2021). Randomized controlled trial assessing the efficacy of expressive writing in reducing anxiety in first-year college students: the role of linguistic features. *Psychology & health*, *36*(9), 1041-1065.
3rd person singular	she, her, him	
<u>*Psychological processes*</u><br><br><u>*(Studies quoted here used all the sub- categories listed below)*</u>		Lumontod III, R. Z. (2020). Seeing the invisible: Extracting signs of depression and suicidal ideation from college students' writing using LIWC a computerized text analysis. *Int. J. Res. Stud. Educ*, *9*, 31-44.<br><br>Low, D. M., Rumker, L., Talkar, T., Torous, J., Cecchi, G., & Ghosh, S. S. (2020). Natural language processing reveals vulnerable mental health support groups and heightened health anxiety on reddit during covid-19:

		Observational study. *Journal of medical Internet research*, *22*(10), e22635.<br><br>Varma, P., Burge, M., Meaklim, H., Junge, M., & Jackson, M. L. (2021). Poor sleep quality and its relationship with individual characteristics, personal experiences and mental health during the COVID-19 pandemic. *International journal of environmental research and public health*, *18*(11), 6030.
Affective processes	Happy, cried	Jelinek, L., Stockbauer, C., Randjbar, S., Kellner, M., Ehring, T., & Moritz, S. (2010). Characteristics and organization of the worst moment of trauma memories in posttraumatic stress disorder. *Behaviour Research and Therapy*, *48*(7), 680-685.<br><br>Robertson, S. M., Short, S. D., Sawyer, L., & Sweazy, S. (2021). Randomized controlled trial assessing the efficacy of expressive writing in reducing anxiety in first-year college students: the role of linguistic features. *Psychology & health*, *36*(9), 1041-1065.
Positive emotion	Love, nice, sweet	Tov, W., Ng, K. L., Lin, H., & Qiu, L. (2013). Detecting well-being via computerized content analysis of brief diary entries. *Psychological assessment*, *25*(4), 1069.<br><br>Desrosiers, A., Vine, V., & Kershaw, T. (2019). "RU Mad?": Computerized text analysis of affect in social media relates to stress and substance use among ethnic minority emerging adult males. *Anxiety, Stress, & Coping*, *32*(1), 109-123.
Negative emotion	Hurt, ugly, nasty	Tov, W., Ng, K. L., Lin, H., & Qiu, L. (2013). Detecting well-being via computerized content analysis of brief diary entries. *Psychological assessment*, *25*(4), 1069.<br>Desrosiers, A., Vine, V., & Kershaw, T. (2019). "RU Mad?": Computerized text analysis of affect in social media relates to stress and substance use among ethnic minority emerging adult males. *Anxiety, Stress, & Coping*, *32*(1), 109-123.
Social processes	Mate, talk, daughter, buddy	
Cognitive processes	Cause, know, ought	Jelinek, L., Stockbauer, C., Randjbar, S., Kellner, M., Ehring, T., & Moritz, S. (2010).

		Characteristics and organization of the worst moment of trauma memories in posttraumatic stress disorder. *Behaviour Research and Therapy*, *48*(7), 680-685.
Biological processes<br><br>*(Studies quoted here used all the sub- categories listed below)*	Eat, blood, pain	Low, D. M., Rumker, L., Talkar, T., Torous, J., Cecchi, G., & Ghosh, S. S. (2020). Natural language processing reveals vulnerable mental health support groups and heightened health anxiety on reddit during covid-19: Observational study. *Journal of medical Internet research*, *22*(10), e22635.
Health	Clinic, flu, pill	
Time orientation<br><br>*(Studies quoted here used all the sub- categories listed below)*		Jelinek, L., Stockbauer, C., Randjbar, S., Kellner, M., Ehring, T., & Moritz, S. (2010). Characteristics and organization of the worst moment of trauma memories in posttraumatic stress disorder. *Behaviour Research and Therapy*, *48*(7), 680-685.
Past focus	Ago, did, talked	
Present Focus	Today, is, now	
Future Focus	May, will, soon	

Table S2. Follow up regression models follow-up models with added predictors (IES and DASS scores).

	**β Coefficient**	***p***	**95% CI**
General Affect			
Cognitive Reserve	-.23	<.001	-.30 – -.15
IES total score	-.08	.004	-.14 – -.03
DAAS Depression	.28	.006	.08 – .48
DAAS Anxiety	-.96	<.001	-1.24 – -.67
DAAS stress	.58	<.001	.45 – .70
Positive Emotions			
Cognitive Reserve	-.07	<.001	-.08 – -.07
IES total score	-.08	<.001	-.08 – -.07
DAAS Depression	-.21	<.001	-.23 – -.19
DAAS Anxiety	.23	<.001	.21 – .26
DAAS stress	-.04	<.001	-.06 – -.03
Negative Emotions			
Cognitive Reserve	-.22	<.001	-.30 – -.14
IES total score	-.02	.51	-.07 – .04
DAAS Depression	.66	<.001	.46 – .86
DAAS Anxiety	-1.23	<.001	-.152 – -.95
DAAS stress	.31	<.001	.18 – .43
Anxiety			
Cognitive Reserve	.17	<.001	.15 – .19
IES total score	.14	<.001	.12 – .15
DAAS Depression	-.24	<.001	-.29 – -.18
DAAS Anxiety	.18	<.001	.10 – .26
DAAS stress	.11	<.001	.08 – .15
Sadness			
Cognitive Reserve	-.09	<.001	-.12 – -.06
IES total score	-.01	.62	-.03 – .02
DAAS Depression	.07	.06	-.01 – .15
DAAS Anxiety	-.31	<.001	-.42 – -.20
DAAS stress	.29	<.001	.24 – .34
Affiliation			
Cognitive Reserve	.11	<.001	.06 – .16
IES total score	-.18	<.001	-.22 – -.14
DAAS Depression	.12	.07	-.02 – .26
DAAS Anxiety	.36	<.001	.16 – .55
DAAS stress	-.10	.02	-.19 – -.02
Achievement			

Cognitive Reserve	.21	<.001	.19 – .22
IES total score	.10	<.001	.09 – .11
DAAS Depression	-.50	<.001	-.54 – -.46
DAAS Anxiety	.60	<.001	.54 – .67
DAAS stress	.19	<.001	.16 – .21
Reward			
Cognitive Reserve	-.14	<.001	-.17 – -.11
IES total score	-.22	<.001	-.24 – -.20
DAAS Depression	.43	<.001	.35 – .50
DAAS Anxiety	-.22	<.001	-.32 – -.11
DAAS stress	-.21	<.001	-.26 – -.16
Risk taking			
Cognitive Reserve	.08	<.001	.06 – .09
IES total score	.04	<.001	.02 – .04
DAAS Depression	-.41	<.001	-.44 – -.38
DAAS Anxiety	.63	<.001	.58 – .68
DAAS stress	-.03	.01	-.05 – -.01
Focus on the past			
Cognitive Reserve	-.09	<.001	-.11 – -.08
IES total score	-.06	<.001	-.07 – -.05
DAAS Depression	.04	.01	.01 – .07
DAAS Anxiety	-.10	<.001	-.14 – -.05
DAAS stress	-.07	<.001	-.09 – -.05
Focus on the present			
Cognitive Reserve	.11	.01	.04 – .19
IES total score	-.01	.80	-.06 –.05
DAAS Depression	.15	.13	-.05 – .35
DAAS Anxiety	-.10	.47	-.39 – .18
DAAS stress	.03	.60	-.09 – .16
Focus on the future			
Cognitive Reserve	.53	<.001	.50 – .56
IES total score	.07	<.001	.05 – .09
DAAS Depression	-.40	<.001	-.48 – -.33
DAAS Anxiety	1.44	<.001	1.34 – 1.55
DAAS stress	-.50	<.001	-.55 – -.45
